# Molecular Surveillance of Enteric Pathogen-Associated Genetic Markers Across Household Environmental Matrices: A Pilot Study in an Urban Informal Settlement in Johannesburg, South Africa

**DOI:** 10.3390/pathogens15101038

**Published:** 2026-10-01

**Authors:** Kavani Sanasi, Tobias George Barnard, Kulsum Kondiah, Atheesha Singh

**Affiliations:** 1 Water and Health Research Centre, Faculty of Health Sciences, University of Johannesburg, PO Box 17011, Doornfontein 2028, South Africa; ksanasi@gmail.com (K.S.); tgbarnard@uj.ac.za (T.G.B.); 2 Department of Biotechnology and Food Technology, University of Johannesburg, PO Box 17011, Doornfontein 2028, South Africa; kulsumk@uj.ac.za

**Keywords:** WASH, environmental surveillance, *Escherichia coli*, *Vibrio* spp., m-PCR

## Abstract

Household environments in informal settlements may serve as potential environmental reservoirs or contamination points and transmission interfaces for enteric pathogens, particularly where water, sanitation, and hygiene (WASH) services are inadequate. Conventional WASH monitoring often focuses on infrastructure and source-water quality, providing limited information regarding potential household-level exposure pathways. A pilot cross-sectional study was conducted in Zandspruit, an informal settlement in Johannesburg, South Africa, using a multi-matrix environmental sampling approach. A total of 143 samples, including drinking water (*n* = 29), dishcloths (*n* = 28), Bio-Wipes of hand (*n* = 29), surfaces (*n* = 28), and toilet seats (*n* = 29), were collected from participating households (*n* = 30). Physicochemical water-quality parameters, according to the South African National Standard (SANS) 241: 2015 by the South African Bureau of Standards (SABS), and microbial indicators were assessed. Deoxyribonucleic acid (DNA) was extracted using a silica-based method, and multiplex polymerase chain reaction (m-PCR) assays were used to detect *Escherichia coli* (*E. coli*) and *Vibrio* species (spp.) from environmental samples. The measured physicochemical parameters were generally within the applicable national standards guideline ranges; however, enteric pathogen-associated genetic markers were detected in selected water samples. Molecular analysis demonstrated marked differences in the occurrence of *E. coli* pathotype-associated markers across household environmental matrices. ETEC was detected in drinking water (5/29, 17.2%) and was most frequent in dishcloths (12/28, 42.9%), followed by surfaces (10/28, 35.7%) and hands (9/29, 31.0%). STEC was detected in hands (10/29, 34.5%), surfaces (9/28, 32.1%), dishcloths (6/28, 21.4%), and toilets (6/29, 20.7%), but not in water. Matrix-specific associations were observed for STEC, typical EPEC, ETEC, and *astA*-positive *E. coli* after Benjamini–Hochberg correction. *Vibrio*-associated markers were detected occasionally: *wbeO* in one dishcloth (1/28, 3.6%) and *sodB* in one hand (1/29, 3.4%) and one surface sample (1/28, 3.6%); *ctxA* was not detected. These findings indicate that household contact matrices may represent important points of molecular contamination and potential cross-contamination. Molecular approaches such as Polyemerase Chain Reaction (PCR) does not establish viability, infectivity, toxin expression, or transmission, therefore the findings should not be interpreted as evidence of viable pathogens or disease. Longitudinal studies incorporating culture-based confirmation and quantitative molecular approaches are needed. Nevertheless, the results support the importance of household hygiene interventions targeting hands, dishcloths, and frequently touched surfaces.

## 1. Introduction

Safe water, sanitation, and hygiene (WASH) systems are necessary to prevent enteric disease transmission. However, in many low-income and informal settings, these systems remain inconsistent, overstretched, or incomplete, increasing household vulnerability to environmental contamination and daily exposure risks [1,2]. Rapid population growth, intermittent water supply, inadequate sanitation infrastructure, and poor waste management place increasing pressure on already fragile systems, forcing households to adopt alternative strategies for water collection, storage, and sanitation management [3]. In many informal settlements (non-permanent dwelling structures not linked to water and sanitation services) across sub-Saharan Africa, people depend on shared sanitation facilities, communal water points, and informal drainage systems, creating complex transmission pathways that are not always adequately captured by conventional WASH monitoring approaches [4].

Current global WASH frameworks mainly focus on infrastructure and source-water quality, with limited attention given to contamination occurring within households, which is where main human exposure takes place [5,6]. Household environments may function as potential environmental reservoirs or contamination points and transmission interfaces where pathogens persist on surfaces, water-storage containers, food preparation areas, and on frequently handled objects, with inadequate WASH infrastructure [7,8]. Routine activities such as water storage, food preparation, cleaning, childcare, and sanitation practices may therefore potentially facilitate the persistence and transfer of enteric pathogens within these domestic environments.

WASH research in South Africa has historically focused on infrastructure, governance, and service access, with partial emphasis on household-level microbiological surveillance. which captures environmental and behavioural determinants of exposure [9]. Recent waterborne disease outbreaks further highlight the importance of environmental surveillance within vulnerable communities. South Africa experienced a major cholera outbreak in Hammanskraal, near Johannesburg, during 2023, resulting in more than 99 confirmed cases and multiple fatalities. Investigations also reported elevated levels of faecal contamination and widespread *E. coli* contamination in rivers, and wastewater-impacted environments [10,11]. These events underscore the continuing vulnerability of communities with compromised WASH infrastructure, highlighting the need for improved approaches to environmental pathogen surveillance.

*Escherichia coli* is an indicator of faecal pollution, is a facultative anaerobic, Gram-negative, usually rod-shaped bacterium [12], and is well studied due to its colonisation of the gut (commonly known as a gut microbe) of warm-blooded animals [13]. This has routinely been used to monitor water quality [14]. *E. coli*, which naturally occurs in the intestine, is mostly commensal. However, when it contains genetic elements that encode for virulence factors aiding in pathogenesis, the bacteria are known to be pathogenic [15,16].

Pathogenic *E. coli* is an important etiological agent that results, among others, in urinary tract infections and intestinal disease, which leads to mild to severe forms of diarrhoea [17,18]. Strains that have been identified to cause gastrointestinal illnesses (diarrhoeagenic *E. coli*) include: enterohaemorrhagic *E. coli* (EHEC), enterotoxigenic *E. coli* (ETEC), enteropathogenic *E. coli* (EPEC), enteroinvasive *E. coli* (EIEC) and enteroaggregative *E. coli* (EAEC) [19,20]. Pathogenic *E. coli* strains are characterised by the detection of virulence genetic markers of each pathotype [21]. Molecular methods such as polymerase chain reaction (PCR) and subsequent sequencing offer greater speed, sensitivity, and specificity for identifying and characterising pathogenic strains compared to traditional culture-based approaches. Preference for non-culturable methods is also due to their sensitivity, reliability, and cost-effectiveness [22]. Further to this, multiplex PCR (mPCR) is utilised to determine the presence or absence of these pathogens. This approach allows for simultaneous detection of multiple virulence genes, thereby saving time required for analysis and results [23]

Diarrhoeagenic *E. coli* (DEC) comprises genetically and phenotypically diverse pathotypes that differ in their virulence mechanisms, clinical manifestations, and epidemiological importance. Molecular detection of pathotype-associated virulence genes provides an important means of identifying DEC directly from clinical specimens; however, individual enteric pathogen-associated genetic markers may not be pathotype-specific, and their detection does not necessarily establish viable organisms or clinical causation. The principal DEC pathotypes relevant to the present study are described below.

Typical enteropathogenic *E. coli* (tEPEC) causes attaching and effacing (A/E) lesions through the coordinated action of the locus of enterocyte effacement, including the type III secretion system and intimin encoded by *eae*. Unlike atypical EPEC, tEPEC additionally carries the EPEC adherence factor plasmid encoding bundle-forming pilus (*bfp*), which facilitates initial adherence and bacterial aggregation [24,25,26]. tEPEC is an established cause of diarrhoeal disease, especially among young children, and has important implications for enteric disease surveillance. The detection of a *BFP-associated* target is consistent with the presence of genetic material characteristic of tEPEC, although molecular detection alone does not establish organism viability or clinical causation.

Atypical enteropathogenic *E. coli* (aEPEC) possesses the A/E-associated *eae* gene but lacks the EAF plasmid and characteristic BFP of tEPEC [24,25,26]. aEPEC has been associated with both acute and persistent diarrhoeal disease, particularly in children, although its epidemiological significance is complicated by substantial genetic heterogeneity and detection in asymptomatic individuals. Importantly, *eae* is also present in other *E. coli* lineages, including STEC/EHEC; therefore, detection of *eae* alone should not be interpreted as definitive evidence of aEPEC.

*Shiga* toxin-producing *E. coli* (STEC)/enterohaemorrhagic *E. coli* (EHEC), where STEC is defined by the presence of *Shiga* toxin genes (stx1 and/or stx2), with some strains additionally carrying the LEE pathogenicity island and producing A/E lesions [25,27]. STEC infections are of particular clinical and public-health importance because they can cause diarrhoea and haemorrhagic colitis and may progress to haemolytic uraemic syndrome (HUS) [28]. Detection of a *stx* target therefore represents a clinically and epidemiologically important finding and may warrant further characterisation for surveillance or outbreak investigation. Nevertheless, *stx* detection alone indicates the presence of *Shiga* toxin-associated genetic material and does not necessarily demonstrate viable STEC or define the infecting strain.

Enteroinvasive *E. coli* (EIEC) produces disease through invasion of intestinal epithelial cells, using mechanisms that closely resemble those of *Shigella* spp. [24,25]. Infection is associated with inflammatory and dysentery-like diarrhoea and is transmitted mainly through the faecal–oral route. Detection of an invasion-associated target such as *ial* provides molecular evidence of an invasive *E. coli* phenotype; however, interpretation may require caution because of the close genetic relationship between EIEC and *Shigella* [29].

Enteroaggregative *E. coli* (EAEC) is characterised by aggregative adherence, biofilm formation and the production of multiple virulence-associated factors. The transcriptional regulator *aggR* is a commonly used molecular marker, although EAEC is genetically heterogeneous and no single virulence determinant is universally present [24,25]. EAEC is associated with acute and persistent diarrhoea and has epidemiological relevance in childhood diarrhoeal disease and travellers’ diarrhoea. Detection of an EAEC-associated target, including the *eagg* target, indicates the presence of EAEC-associated genetic material but should be interpreted in conjunction with clinical and epidemiological findings.

Enterotoxigenic *E. coli* (ETEC) causes secretory diarrhoea through production of heat-labile and/or heat-stable enterotoxins encoded by *lt* and *st*, respectively [24,25]. ETEC is an important cause of childhood diarrhoeal disease in low- and middle-income countries and is also a major cause of travellers’ diarrhoea, with transmission strongly influenced by sanitation, water quality and food hygiene. Detection of *lt* and/or *st* therefore provides molecular evidence of an enterotoxin-associated ETEC genotype, although the presence of a virulence-associated gene alone does not establish that ETEC is responsible for an individual’s illness [30].

*Escherichia coli* and *Vibrio* spp. were selected as the primary bacterial targets because of their epidemiological significance, regulatory relevance, and established association with WASH-related disease risk. Studies conducted within Gauteng informal settlements have demonstrated elevated concentrations of *E. coli* and other faecal indicator bacteria associated with inadequate sanitation, wastewater overflows, and contaminated surface runoff [10]. *Vibrio* spp., particularly *V. cholerae*, remain important public health pathogens in South Africa. Together, these organisms provide indicators of both general faecal contamination and pathogen-specific disease risk.

Zandspruit is an informal settlement located in the Gauteng Province of South Africa. It was selected as the pilot study site because it exhibits many characteristics associated with WASH vulnerability, including rapid population growth, limited sanitation infrastructure, intermittent water supply, and reliance on communal sanitation facilities. Inconsistent access to potable water, shared sanitation facilities, inadequate waste management, and close interaction between households and the surrounding environment create multiple pathways for microbial exposure that cannot be adequately assessed through infrastructure-based indicators or source-water monitoring alone [4,31].

This pilot study therefore employed a multi-matrix household sampling approach to investigate enteric pathogen contamination within a WASH-constrained informal settlement. Drinking water, dishcloths, and Bio-Wipe samples collected from hands, domestic surfaces, and toilet seats were analysed to adapt and develop a practical, field-based protocol for the recovery and detection of low-level enteric pathogen-associated genetic markers. By integrating environmental sampling across multiple household matrices, the study aimed to provide a more comprehensive understanding of the presence or absence of enteric bacterial pathogens within domestic environments and contribute evidence to support context-specific WASH interventions and household-level environmental surveillance strategies.

## 2. Materials and Methods

### 2.1. Study Design and Site Description

This study employed an exploratory cross-sectional design based on a point-in-time assessment to molecularly survey enteric pathogen-associated genetic markers across household environmental matrices within selected informal households in Zandspruit, Johannesburg, South Africa. Informal settlements often differ from formal residential areas with respect to building materials, service delivery, and environmental conditions, making them important settings for investigating household-level environmental contamination.

**Figure 1:**
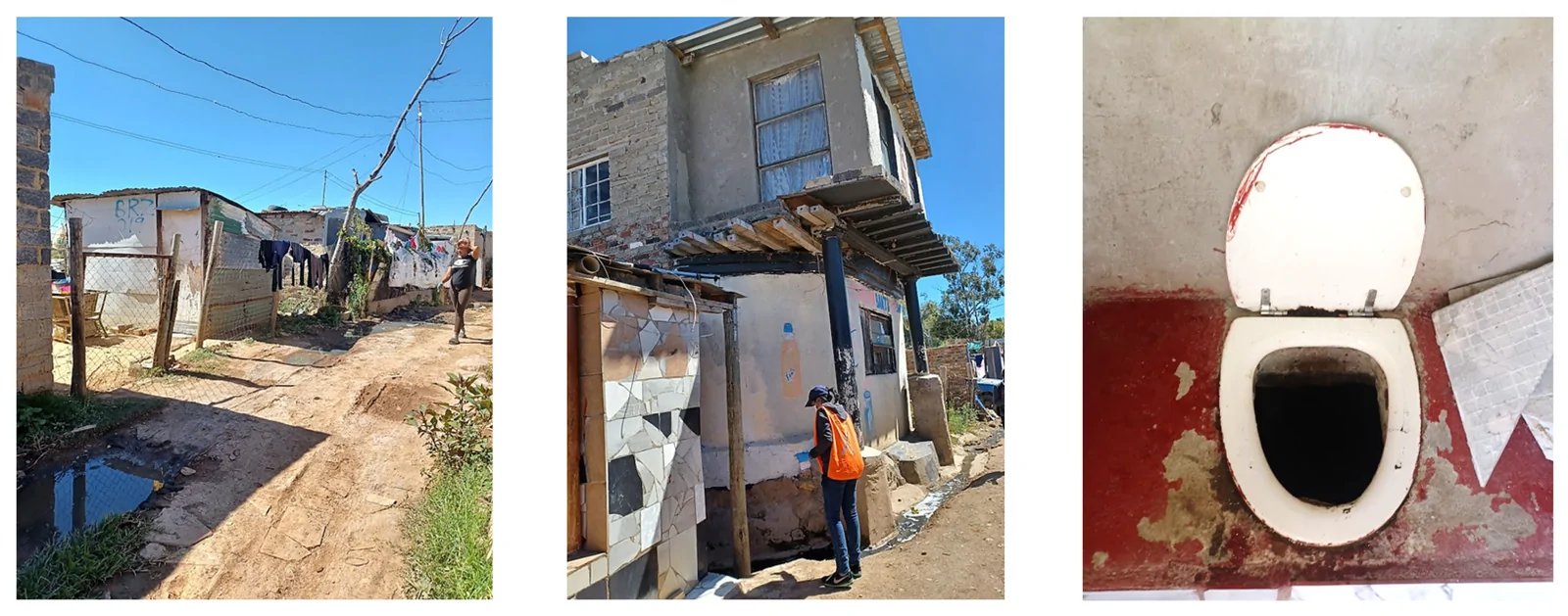
Zandspruit Sampling Site showing the informal housing structures; poorly and often illegally connected electrical wiring; and sanitation infrastructure, often with dilapidated ceilings, stagnant water, and dirt outside pit latrine toilets that have unmounted toilet seats.

Zandspruit is an informal settlement (Figure 1) established in 1994 and is in Johannesburg within the Gauteng Province of South Africa. The settlement developed through informal occupation on land that was previously privately owned farmland, resulting in spatial heterogeneity with areas that are variably developed and underdeveloped [4,31]. Informal settlements of this nature are characteristic of rapid urbanisation processes observed across major South African metropolitan areas [32]. Approximately 43.8% of dwellings have intermittent access to basic services and consist of corrugated iron shacks, consistent with housing patterns reported in other informal settlements within the Gauteng Province [32].

### 2.2. Participant Recruitment and Ethics

Ethical clearance for sample collection and analysis was granted by the Faculty of Health Research Ethics Committee (REC-1729-2022) at the University of Johannesburg. Household participation was based on voluntary recruitment and informed consent, consistent with approved ethical procedures. Consequently, the sampled households (*n* = 30) represent a convenience sample rather than a statistically representative population. This approach was adopted to facilitate preliminary assessments of environmental contamination within households and is consistent with previous environmental microbiology and WASH studies conducted in informal settlements [7,8].

Prior to household recruitment and sampling at the site, community leaders were contacted for approval, and once approval was obtained, residents were contacted for permission based on the convenience sampling method, whereby residents from households that were present were asked to participate in the study. Once these residents agreed to be participants, they were asked to complete consent forms and were given the information sheet to retain.

The study involved recruitment of consenting human household participants for environmental sampling, which included the collection of samples from participants’ hands, but did not involve clinical intervention, experimental treatment, or collection of clinical diagnostic specimens.

No structured behavioural or hygiene questionnaire was administered; therefore, the study could not assess associations between household hygiene practices and environmental contamination.

### 2.3. Environmental Sample Collection and Processing

Sampling was conducted according to household availability and participant consent. During 2022, environmental samples collected from Zandspruit included drinking water (*n* = 29), dishcloths (*n* = 28), hand Bio-Wipe samples (*n* = 29), domestic surface Bio-Wipe samples (*n* = 28), and toilet-seat Bio-Wipe samples (*n* = 29). Samples were transported to the Water and Health Research Centre (WHRC), University of Johannesburg, in insulated cooler boxes and processed within 48 h of collection. Remaining sample aliquots were preserved at −20 °C in 50% (*v*/*v*) glycerol for subsequent analyses.

#### 2.3.1. Drinking Water Sampling

One-litre municipal drinking water samples were collected aseptically in autoclaved Schott bottles containing 1% sodium thiosulphate to neutralise residual chlorine. Sampling included tap or standpipe water depending on availability. Physico-chemical parameters were measured in situ. Total coliform and *E. coli* detection was performed using IDEXX Colilert^®^ (IDEXX Laboratories (Pty) Ltd., Johannesburg, South Africa) and the Most Probable Number (MPN) method, following the manufacturer’s guidelines.

#### 2.3.2. Physical and Chemical Drinking Water Quality

Drinking water was tested for physico-chemical parameters. Temperature (°C), the potential of hydrogen ions (pH), and conductivity (µS/cm) were measured on-site using a portable Combo Tester^®^ (Hanna, Sigma-Aldrich, Saint Louis, MI, USA). Residual chlorine levels were measured on-site using a chlorine photometer (Hanna Instruments, Johannesburg, South Africa) according to the manufacturer’s instructions. Total Dissolved Solids (TDS) was not measured and therefore could not be evaluated as part of the physico-chemical characterisation of household drinking water. However, the parameters measured in this study were selected to address the primary objectives concerning water quality and microbial contamination. All equipment was calibrated according to the manufacturers’ instructions. Turbidity was measured using the portable 2100P turbidimeter) (Hach South Africa (Pty) Ltd., Johannesburg, South Africa) and the TN100 EUTECH turbidimeter at the WHRC laboratory.

#### 2.3.3. Microbiological Drinking Water Quality

Drinking water was tested for *E. coli* and total coliform contamination using the most probable number (MPN) approach. Drinking water was analysed with the IDEXX Colilert^®^ Quanti-Tray™ (IDEXX Laboratories Inc., Pretoria, South Africa), upon arrival at the laboratory. *Klebsiella pneumonia* strain (ATCC No.31488) was used as a standard coliform-positive control, forming yellow-coloured wells; *Pseudomonas aeruginosa* strain (ATCC No. 27853), a non-coliform, served as a negative control, remaining colourless. A positive control for *E. coli* (ATCC No. 25922) and ddH20 was used as a product control. The Quanti-Trays^®^ were examined under long-wave 366 nanometre (nm) ultraviolet light (UV). Wells that turned yellow and did not fluoresce were positive for Total coliforms, and wells that turned both yellow and fluoresced were *E. coli* positive (IDEXX) and recorded as MPN/100 millilitre (mL) of water sample and interpreted using the table supplied by the manufacturer. Positive samples were used for nucleic acid extraction and downstream molecular analyses.

#### 2.3.4. Bio-Wipe Sampling

Environmental sampling was performed using the Bio-Wipe system originally developed for enteric pathogen collection [33,34]. The system consists of a permeable Bio-Wipe used in conjunction with polyester batting material to facilitate microbial recovery. As the Bio-Wipe system was originally designed for stool collection, methodological adaptations were undertaken to optimise environmental sampling. Bio-Wipes were premoistened with 5 mL phosphate-buffered saline (PBS) prior to sampling. Samples were collected from participants’ hands, toilet seats, and frequently touched household surfaces. Approximately 30 × 60 cm of surface area was sampled where possible. Following sample collection, wipes were transported in modified Cary-Blair transport medium (1.5 g Sodium Thioglycolate, 2.09 g Na_2_HPO_4_. 7H_2_O, 5.0 g NaCl, 1% 3.2 mL CaCl_2_, 2.5 g Agar). The samples were processed within 48 h. Microbial material was recovered using 10 mL PBS (Sigma) containing 0.1% Tween 20 (Atlas Chemie, Essen, Germany), and the resulting eluates were used for nucleic acid extraction and downstream molecular analyses.

#### 2.3.5. Dishcloth Sampling

Used household dishcloths were collected and replaced with new cloths. In the laboratory, 5 × 5 cm sections were aseptically excised and immersed in 10 mL PBS containing 0.1% Tween 20. Samples were incubated for 10 min with intermittent vortexing to facilitate microbial recovery. Aliquots of the resulting suspension were used for nucleic acid extraction and downstream molecular analyses.

### 2.4. DNA Extraction

Bacterial DNA was extracted using an in-house silica-column method routinely employed at the WHRC and adapted from the methods outlined in [35,36,37]. Briefly, using the guanidium thiocyanate method [23], samples were subjected to lysis, ethanol precipitation, celite adsorption, column purification, washing, and elution steps to obtain purified bacterial DNA. The supernatant was pipetted into 1.5 mL Eppendorf tubes as a DNA sample and kept at −20 °C for downstream processing.

The DNA concentration and purity were measured spectrophotometrically using a NanoPhotometer NP60 (Implen GmbH, Munich, Germany). Absorbance ratios at 260/280 nm and 260/230 nm were used to evaluate nucleic acid purity.

### 2.5. Multiplex-Polymerase Chain Reaction (m-PCR)

Primer sets targeting *E. coli* and *Vibrio* spp. were synthesised commercially (Inqaba Biotechnologies and Whitehead Scientific, Pretoria, South Africa). Primer specificity was verified using the National Centre for Biotechnology Information Basic Local Alignment Search Tool (NCBI BLAST v5) database before use. Primer sequences and expected amplicon sizes are provided in Table 1.

#### 2.5.1. Bacterial Controls and Assay Validation

Control bacterial strains were obtained from the American Type Culture Collection (ATCC). *Escherichia coli* ATCC 25922 was used as a positive control for *E. coli* amplification, and the housekeeping *mdh* gene that encodes for malate dehydrogenase, an enzyme involved in the citric acid cycle (Krebs cycle), was included as an internal control. Positive controls for *Vibrio* spp. consisted of DNA extracted from both pathogenic and commensal *Vibrio* reference strains maintained at the Water and Health Research Centre (WHRC), originally obtained from the National Health Laboratory Service and the National Collection of Type Cultures (NCTC). Amplification of the bacterial 16S rRNA gene was included to confirm the presence and integrity of bacterial DNA and to assess PCR performance. Sterile PCR-grade water was used as the negative control.

The PCR assays targeting enteric pathogen-associated genetic markers in *Escherichia coli* and *Vibrio* spp. were based on previously published and validated methodologies [33,38,39,40]. The same or corresponding validated primer sets and established positive-control strains used in previous studies conducted within the same research laboratory were used in the present study. These controls had been validated previously for the relevant target genes; target-specific sequence confirmation was not repeated in the present study. and shown in Table 1.

**Table 1:** *E. coli* and *Vibrio* spp. primers used for m-PCR.

Pathogen	Gene	Primer Sequence (5′-3′)	Size (bp)	Reference
***Escherichia coli***				
Internal Control	*mdh (F)*	GGT ATG GAT CGT TCC GAC CT	304	[41]
	*mdh (R)*	GGC AGA ATG GTA ACA CCA GAG T		
*EIEC*	*ial (F)*	GGT ATG AGT CCA	650	[42]
	*ial (R)*	GGA GGC CAA TTA TTT CC		
*EHEC* *Atypical EPEC*	*eaeA (F)*	CTG AAC GGC GAT TAC GCG AA	917	[43]
	*eaeA (R)*	CCA GAC GAT ACG ATC CAG		
*Typical EPEC*	*bfpA (F)*	AAT GGT GCT TGC GCT TGC	410	[43]
	*bfpM (R)*	TAT TAA CAC CGT AGC CTT TCG CTG AAG TAC CT		[23]
*EAEC*	*eagg (F)*	AGA CTC TGG CGA AAG ACT GTA TC	194	[44]
	*eagg (R)*	ATG GCT GTC TGT AAT AGA TGA GAA C		
*EHEC*	*stx1 (F)*	ACA CTG GAT GAT CTC AGT GG	614	[45]
	*stx1 (R)*	CTG AAT CCC CCT CCA TTA TG		
	*stx2 (F)*	CCA TGA CAA CGG ACA GCA GTT	779	[45]
	*stx2 (R)*	CCT GTC AAC TGA GCA CTT TG		
*ETEC*	*lt (F)*	TGG ATT CAT GCA CCA CAA GG	360	[44]
	*lt (R)*	CCA TTT CTC TTT TGC CTG CCA TC		
	*st (F)*	TTT CCC CTC TTT TAG TCA GTC AAC TG	160	[44]
	*st (R)*	GGC AGG ATT ACA AAG TTC ACA		
*E. coli toxin*	*astA (F)*	GCC ATC AAC ACA GTA TAT CC	106	[46]
	*astA (R)*	GAG TGA CGG CTT TGT AGT C		
External Control	*gapdh (F)*	GAG TCA ACG GAT TTG GTC GT	238	[47]
	*gapdh (R)*	TTG ATT TTG GAG GGA TCT CG		
***Vibrio* spp.**				
Internal Control	*16S (F)*	CGG TGA AAT GCG TAG AGA T	663	[48]
	*16S (R)*	TTA CTA GCG ATT CCG AGT TC		
*V. cholerae O1*	*wbeO (F)*	GTT TCA CTG AAC AGA TGG G	192	[49]
	*wbeO (R)*	GGT CAT CTG TAA GTA CAA C		
*V. cholerae spp*	*sodB (F)*	AAG ACC TCA ACT GGC GGT A	248	[48]
	*sodB (R)*	GAA GTG TTA GTG ATC GCC AGA GT		
*Cholera toxin A*	*ctxA (F)*	ACA GAG TGA GTA CTT TGA CC	308	[49]
	*ctxA (R)*	ATA CCA TCC ATA TAT TTG GGA G		

These positive controls had previously been validated for the relevant target genes. Positive and negative controls were included in the PCR assays to assess amplification performance and monitor potential contamination. PCR products were resolved by agarose gel electrophoresis and evaluated according to the expected amplicon sizes reported for the respective primer sets. The *mdh* and 16S rRNA targets were used as internal amplification controls for *E. coli* and *Vibrio* spp., respectively, to assess bacterial DNA integrity and amplification competence. Collectively, the use of previously validated primer sets and positive controls, together with appropriate negative and internal amplification controls and assessment of expected amplicon sizes, provided quality assurance for detection of the targeted pathogen-associated genetic markers.

*E. coli* pathotypes were classified according to predefined combinations of PCR-detected virulence-associated genes rather than on the detection of individual genes in isolation. Classification was applied hierarchically to distinguish overlapping virulence profiles. First, samples positive for *stx1* and/or *stx2* were classified as *Shiga* toxin-producing *E. coli* (STEC), irrespective of the presence or absence of *eae* and *bfp*. Thus, samples carrying *stx* together with *eae* and *bfp* were classified as STEC and not as typical enteropathogenic *E. coli* (tEPEC). For samples negative for both *stx1* and *stx2*, *eae* and *bfp* were subsequently assessed: samples positive for both *eae* and *bfp* were classified as typical EPEC, whereas those positive for *eae* but negative for *bfp* were classified as atypical EPEC. Samples negative for *eae* were not assigned to either EPEC category. Independently of the STEC and EPEC classifications, samples positive for *st* and/or *lt* were classified as enterotoxigenic *E. coli* (ETEC), those positive for *eagg* as enteroaggregative *E. coli* (EAEC), and those positive for *ial* as enteroinvasive *E. coli* (EIEC). Detection of *astA* was reported separately as *astA*-positive *E. coli*. Accordingly, ETEC, EAEC, EIEC, and *astA*-positive *E. coli* classifications were permitted to co-occur with STEC or EPEC classifications where the corresponding gene targets were detected. These classifications represent marker-defined molecular profiles and do not establish viable organisms or clinical pathotypes.

#### 2.5.2. Identification of *Escherichia coli* by Multiplex-Polymerase Chain Reaction (m-PCR)

All PCRs were performed in a BIO-RAD^®^ T100^TM^ thermal Mycycler. *E. coli* DNA sample PCR amplification was done following the 11-gene m-PCR protocol optimised by [23]. This PCR protocol, in which eleven different gene markers are amplified in a single reaction tube, can be used to address faecal contamination situations by identifying specific pathogenic strains of *E. coli* that are often overlooked in traditional testing methods. The samples were amplified in a 20 micro litres (µL) reaction mixture containing 10 µL of the 2× Qiagen^®^ m-PCR Master Mix (Hotstart Taq DNA polymerase, 10× buffer, 2 mM MgCl2 and dNTP mix), 1 µL 5× Q-solution, 4.5 µL of PCR grade water, 2 µL MgCl2, 2 µL of template DNA and 0.5 µL of the primer mix [0.1 micro molar (µM) of *mdh* and *lt-1* primers (Forward (F) and Reverse (R)), 0.2 µM of *ial*, *gapdh*, *eagg*, *astA*, and *bfp* primers (F and R), 0.3 µM of *eaeA* and *stx2* primers (F and R), 0.5 µM of *stx1* and *st-a* primers (F and R)]. The PCR conditions were as follows: enzyme activation at 95 °C for 15 min, denaturation for 94 °C for 45 s (sec), annealing at 55 °C for 45 s, elongation at 68 °C for 2 min (35 cycles), final elongation at 72 °C for 5 min, and hold at 4 °C indefinitely.

#### 2.5.3. Identification of *Vibrio* spp. by Multiplex-Polymerase Chain Reaction (m-PCR)

All PCRs were performed in a BIO-RAD^®^ T100^TM^ thermal Mycycler. The *Vibrio* spp. DNA sample PCR amplification was done following the 4-gene m-PCR protocol previously described by [40]. The samples were amplified in a 13 μL reaction mixture containing 10 μL 2× Qiagen m-PCR Master Mix (Master Mix contains HotStartTaq DNA Polymerase, m-PCR buffer with 2 millimolar (mM) MgCl2, and dNTP Mix); 1 μL 5× Qiagen Q-solution; 2 μL genomic DNA; PCR-grade water; and 0.25 μL of the primer mix (0.5 μL of a 10 mM for each primer). The PCR conditions were as follows: enzyme activation at 95 °C for 15 min, denaturation at 94 °C for 45 s, annealing at 55 °C for 45 s, elongation at 72 °C for 60 s (35 cycles), final elongation at 72 °C for 5 min, and hold at 4 °C indefinitely.

#### 2.5.4. Gel Electrophoresis

The PCR products were separated by gel electrophoresis to determine the presence or absence of enteric bacteria. Briefly, 5 µL of PCR amplicons and two microliters of purple loading dye (Quick-Load^®^, New England BioLabs^®^) were visualised directly by electrophoresis on agarose gels prepared at 2.5% (*w*/*v*) for *E. coli* and 2% (*w*/*v*) for *Vibrio* spp. Agarose (molecular biology grade) for the gels was weighed out using an analytical balance (ADAM Nimbus). All gels ≥ 100 mL contained seven microliters of ethidium bromide (EtBr) (0.5 µg/mL) and Tris-Acetate-EDTA (TAE) running buffer (40 mM Tris acetate, 2 mM EDTA, pH 8.3). Electrophoresis was conducted at 80–100 Volts for 45–90 min, and DNA fragment bands were viewed under UV light (Omega Fluor™ Gel Documentation System) and the Gel Doc™ EZ imaging system (BIORAD, Hercules, CA, USA). The relevant sizes of the DNA fragments were estimated by comparing their electrophoretic mobility to that of a standard 100 bp marker (Quick-Load^®^, New England BioLabs^®^), as well as *E. coli* and *Vibrio* spp. controls that were run for each gel, respectively. For multiplex PCR gel analysis, consistency in result interpretation was maintained throughout. Pathotypes were classified as positive or negative based on visual band intensity and the acceptable margin of error for upper and lower size limits. Environmental samples occasionally produced bands exceeding the expected size range by a few base pairs, which were assessed using standard gel electrophoresis interpretation methods.

### 2.6. Data and Statistical Analysis

The amplified DNA fragment bands were viewed under UV light (Omega Fluor™ Gel Documentation System) and the Gel Doc™ EZ imaging system (BIORAD). Data were captured using [50].

All statistical analyses were performed using the Statistical Consultation Service at the UJ, and WHRC was consulted for statistical analysis.

Chi-square tests of independence (matrix × detection status) were used for each target, substituted with a Monte Carlo Fisher–Freeman–Halton exact *p*-value when expected cell counts were below 5. Cramér’s V is reported as the effect size. A Benjamini–Hochberg (FDR) correction was applied within the *E. coli* set and within the *Vibrio* spp. set. Spearman’s rank correlation was used to compare contamination profiles between matrices, based on the seven independent *E. coli* pathotype categories. Analyses were performed using Python v3.12.6 [51] and Google Colaboratory [52].

## 3. Results

### 3.1. Environmental Sample Collection and Processing

A total of 30 households were targeted for molecular environmental sampling, with multiple sample types collected per household, including drinking water, dishcloths, and Bio-Wipe samples from hands, surfaces, and toilet seats. This sampling framework enabled assessment of multiple potential household exposure points within domestic WASH environments.

### 3.2. Physical, Chemical and Microbiological Quality of Drinking Water

**Figure 2:**
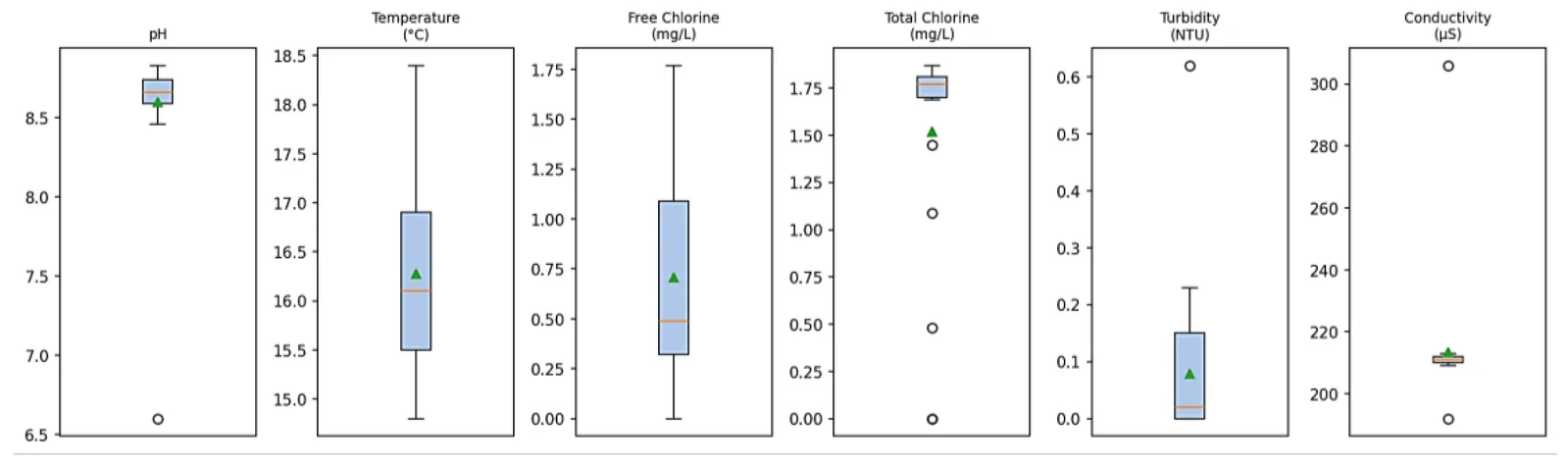
Box-and-whisker plots showing the Physical and Chemical Quality of Drinking Water (*n* = 29) in Zandspruit: pH, Temperature, Free Chlorine, Total Chlorine, Turbidity, Conductivity. The green triangles mark the mean (average) of each parameter’s 29 values—separate from the orange line, which marks the median.

A total of 30 households were targeted in Zandspruit; however, only 29/30 samples were available for analysis, with one sample potentially lost during sample processing and analysis. Drinking water physico-chemical characteristics (Figure 2) were assessed in accordance with SANS 241:2015 [53,54]. The pH ranged from 6.6 to 8.8 (mean 8.67), temperature from 14.8 to 18.4 °C (mean 16.3 °C), free chlorine from 0 to 1.77 mg/L (mean 0.7 mg/L), and total chlorine from 0 to 1.87 mg/L (mean 1.5 mg/L). Turbidity remained low (<0.62 NTU; mean 0.07 NTU), while conductivity ranged from 1.92 to 3.06 µS/cm (mean 2.13 µS/cm).

This is a standard matplotlib box-plot convention (showmeans = True): the box shows the interquartile range (25th–75th percentile), the orange line inside it is the median, and the green triangle is the arithmetic mean plotted on top.

Microbiological quality was assessed using the most probable number (MPN) method for total coliforms and *E. coli*, in accordance with SANS 241:2015, as shown in Table 2.

As shown in Table 2, *E. coli* was absent from all 29/29 (100%) drinking-water samples. Total coliforms were absent from 26/29 (89.7%) samples and detected in 3/29 (10.3%) samples. Of the positive samples, two (6.9%) had total coliform concentrations of 1–10 MPN/100 mL, while one (3.4%) had concentrations of 11–100 MPN/100 mL.

### 3.3. DNA Extraction

DNA was successfully extracted from all environmental matrices, including drinking water, dishcloths, and Bio-Wipe samples collected from hands, surfaces, and toilet seats.

Spectrophotometric quantification at 260 nm demonstrated variation in DNA concentration among sample types. Drinking-water samples yielded the lowest mean DNA concentration (10.39 ± 3.87 ng/µL), whereas toilet-seat samples had the highest mean concentration (27.42 ± 7.37 ng/µL), followed by dishcloth (25.69 ± 6.84 ng/µL), surface (22.69 ± 5.00 ng/µL), and hand samples (18.78 ± 4.75 ng/µL).

Overall, DNA concentrations ranged from 2.97 to 43.87 ng/µL across the sample types. Despite the lower DNA yields obtained from drinking-water samples, the extracted DNA was sufficient for downstream molecular analyses.

### 3.4. Data and Statistical Analysis

Identification of *E. coli* and *Vibrio* spp. by Multiplex-Polymerase Chain Reaction (m-PCR) showing the comparison of detection among household matrices.

The m-PCR assays targeting *E.a coli* pathotypes and *Vibrio* spp. confirmed the presence of multiple enteric pathogen-associated markers across household environmental samples collected in Zandspruit.

Diarrhoeagenic *E. coli* pathotypes were detected across most household environmental targets, although prevalence varied by target (Table 3). No typical EPEC, atypical EPEC, EAEC, EIEC, or *astA*-positive *E. coli* were detected in water samples. Dishcloths showed occurrence of several pathotypes, including STEC (6/28, 21.4%), ETEC (12/28, 42.9%), EAEC (6/28, 21.4%), EIEC (4/28, 14.3%), and *astA*-positive *E. coli* (8/28, 28.6%). Typical EPEC was also detected in dishcloths (4/28, 14.3%).

Hands and household surfaces also showed substantial contamination. STEC was detected in 34.5% of hand samples (10/29) and 32.1% of surface samples (9/28), while ETEC occurred in 31.0% and 35.7%, respectively. EAEC was detected in 20.7% of hand samples and 21.4% of surface samples. Atypical EPEC was detected in 10.3% of hands and 7.1% of surfaces, whereas EIEC was detected in 3.6% of surfaces but not in hands. *AstA*-positive *E. coli* occurred in 17.2% of hand samples and 17.9% of surface samples.

Toilet samples showed STEC (20.7%), atypical EPEC (20.7%), EAEC (17.2%), ETEC (3.4%), EIEC (6.9%), and *astA*-positive *E. coli* (6.9%). Typical EPEC was not detected in hands, surfaces, or toilets. Overall, ETEC was the most frequently detected pathotype, particularly in dishcloths, while STEC showed its highest prevalence in hand samples.

Detection of *astA* was reported separately from the defined diarrhoeagenic *E. coli* pathotypes because the presence of *astA* alone was not considered sufficient to assign a sample to a specific pathotype. Although *astA* encodes enteroaggregative *E. coli* heat-stable enterotoxin 1 (EAST-1), this marker may occur across different *E. coli* genetic backgrounds and was therefore interpreted as an additional virulence-associated marker rather than as a standalone indicator of EAEC. EAEC classification was based on detection of *eagg*, while *astA*-positive samples were reported independently and were permitted to co-occur with other pathotype classifications where the corresponding markers were detected.

**Figure 3:**
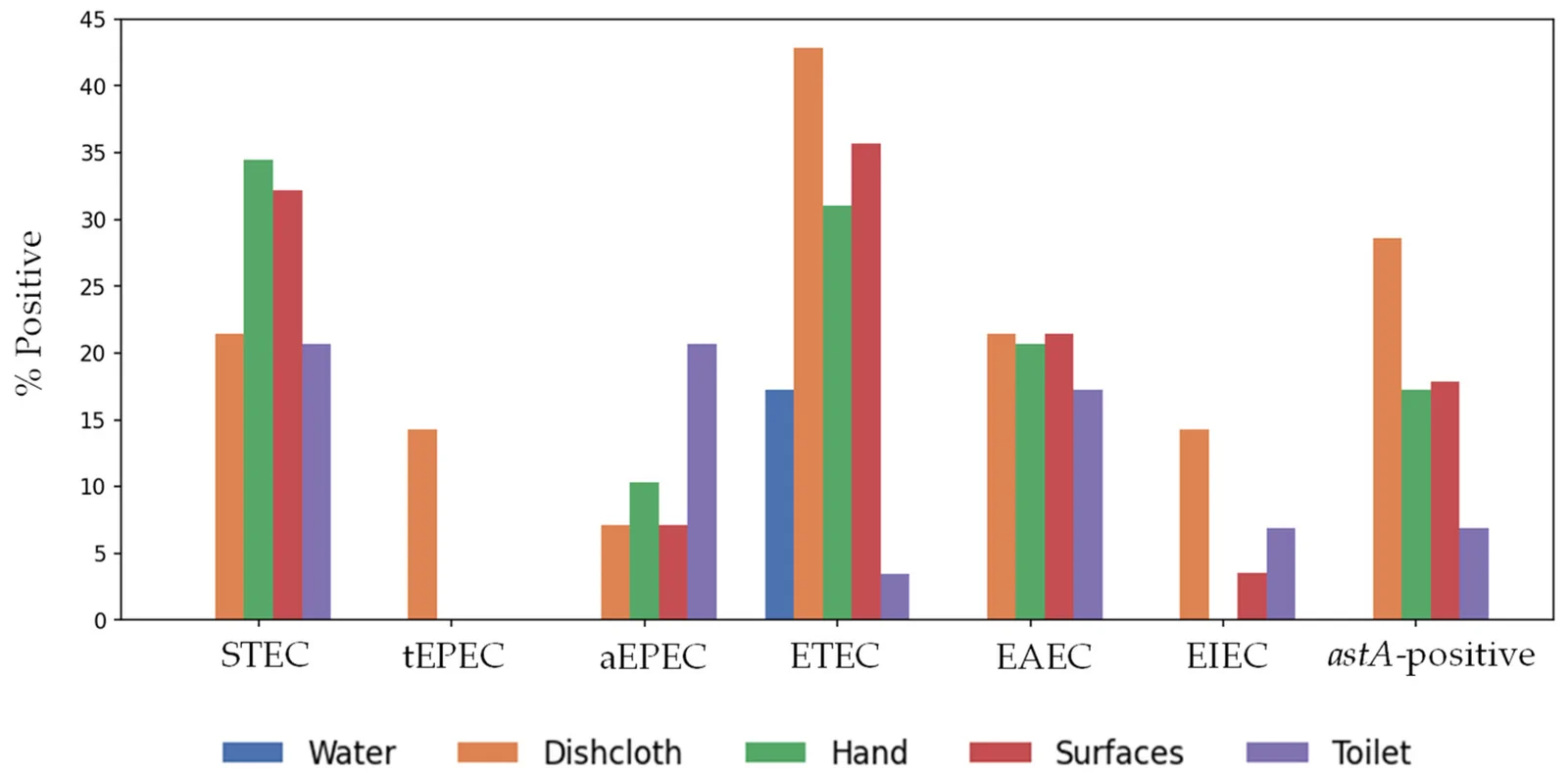
*E. coli* pathotypes prevalence by sample matrix.

Figure 3 illustrates the distribution of *E. coli* pathotypes across the household environmental matrices following classification based on predefined combinations of virulence-associated genes. STEC was detected across all matrices except drinking water, with the highest prevalence in hands (34.5%), followed by household surfaces (32.1%), dishcloths (21.4%) and toilets (20.7%). Typical EPEC (tEPEC) was detected only in dishcloths (14.3%), while atypical EPEC (aEPEC) was detected in dishcloths (7.1%), hands (10.3%), surfaces (7.1%) and toilets (20.7%). ETEC showed the highest overall prevalence among the defined pathotypes, particularly in dishcloths (42.9%), followed by surfaces (35.7%), hands (31.0%) and drinking water (17.2%), but was less frequently detected in toilets (3.4%). EAEC was detected across all non-water matrices, ranging from 17.2% in toilets to 21.4% in dishcloths and surfaces, whereas EIEC was detected primarily in dishcloths (14.3%) and at lower frequencies in surfaces (3.6%) and toilets (6.9%). The *astA* marker was detected in dishcloths (28.6%), hands (17.2%), surfaces (17.9%), and toilets (6.9%), but was not detected in drinking water.

These descriptive patterns were consistent with the matrix-level association analysis presented in Table 4. Significant associations with environmental target were observed for STEC (χ^2^ = 12.65, BH-adjusted *p* = 0.0245; Cramér’s V = 0.297), tEPEC (χ^2^ = 16.90, BH-adjusted *p* = 0.0091; V = 0.334), ETEC (χ^2^ = 14.76, BH-adjusted *p* = 0.0104; V = 0.321), and *astA*-positive *E. coli* (χ^2^ = 11.48, BH-adjusted *p* = 0.0331; V = 0.283). In contrast, aEPEC (BH-adjusted *p* = 0.1218), EAEC (*p* = 0.1614), and EIEC (*p* = 0.0665) were not significantly associated with environmental target after Benjamini–Hochberg correction. The significant tEPEC association should be interpreted in the context of its restricted detection to dishcloths. Overall, the figure demonstrates a broader and more frequent distribution of STEC, ETEC and *astA*-positive *E. coli* across household contact matrices than of tEPEC, aEPEC or EIEC.

Spearman correlation analysis identified positive associations in the distribution of *E. coli* pathotype detection between several environmental matrices (Table 5). The strongest correlation was observed between hands and household surfaces (ρ = 0.27, *p* = 0.554), indicating a very strong and statistically significant positive association. A significant positive correlation was also observed between dishcloths and household surfaces (ρ = 0.76, *p* = 0.046).

Positive, but non-significant, correlations were observed between dishcloths and hands (ρ = 0.67, *p* = 0.098), water and dishcloths (ρ = 0.62, *p* = 0.139), and water and surfaces (ρ = 0.61, *p* = 0.144). Moderate positive correlations were also observed between hands and toilets (ρ = 0.46, *p* = 0.295) and water and hands (ρ = 0.41, *p* = 0.358). Water and toilets showed a moderate negative correlation (ρ = −0.41, *p* = 0.358), although this was not statistically significant.

No evidence of association was observed between dishcloths and toilets (ρ = −0.16, *p* = 0.726), while the correlation between surfaces and toilets was positive but weak-to-moderate and non-significant (ρ = 0.32, *p* = 0.478). Overall, significant correlations were limited to the dishcloth–surface and hand–surface comparisons.

The detection of *Vibrio* spp. and associated virulence markers was generally low across the household environmental matrices examined (Table 6). The serogroup O1-associated *wbeO* marker was detected in only one dishcloth sample (1/28, 3.6%) and was not detected in water, hand, surface, or toilet samples. The species-specific *sodB* marker was detected in one hand sample (1/29, 3.4%) and one surface sample (1/28, 3.6%), but was not detected in water, dishcloth, or toilet samples.

Importantly, the cholera toxin gene *ctxA* was not detected in any of the samples tested, irrespective of the environmental target. Overall, the findings indicate a low occurrence of *Vibrio*-associated molecular markers in the sampled household environments, with detection limited to a small number of dishcloth, hand, and surface samples.

No significant association was observed between environmental target and detection of any individual *Vibrio* marker (Table 7). Detection of the O1-associated *wbeO* marker did not differ significantly among targets (χ^2^ = 4.14, df = 4; BH-adjusted *p* = 0.4569; Cramér’s V = 0.170), nor did detection of the species-specific sodB marker (χ^2^ = 3.06; BH-adjusted *p* = 0.7534; V = 0.146). The *ctxA* marker was not detected in any sample and therefore could not be statistically compared across targets.

Similarly, detection of any *Vibrio* marker (*wbeO*, *sodB*, or *ctxA*) was not significantly associated with environmental target (χ^2^ = 2.09; BH-adjusted *p* = 0.7093; V = 0.121). In contrast, the *Vibrio* 16S internal control showed a strong association with environmental target (χ^2^ = 77.88; df = 4; BH-adjusted *p* < 0.001; Cramér’s V = 0.738), indicating substantial differences in detection of *Vibrio*-associated bacterial DNA among matrices despite the low frequency of specific marker detection.

## 4. Discussion

This study demonstrated microbial contamination across multiple household matrices in Zandspruit, highlighting the household environment as an important component of potential enteric exposure pathways. The application of Bio-Wipes to household surfaces also demonstrated their potential for environmental surveillance beyond their original application in faecal specimen collection [33,34]. Their simple application, minimal equipment requirements, and suitability for field-based sampling may be particularly advantageous in low-resource settings.

The overall findings demonstrate that household environmental matrices differed in the molecular detection of diarrhoeagenic *E. coli* markers. The predominance of detections in dishcloths, hands, and household surfaces is particularly relevant because these matrices represent frequent points of contact and may facilitate transfer of microorganisms within the domestic environment, providing potential exposure pathways [55]. Dishcloths showed both the greatest diversity and relatively high prevalence of several pathotype-associated markers, supporting their potential role as sites of microbial accumulation and transfer. Repeated exposure to moisture, food residues, hands, and multiple surfaces may provide opportunities for persistence and redistribution of microorganisms [56,57,58].

The significant associations between environmental targets and STEC, typical EPEC, ETEC, and *astA*-positive *E. coli*, together with the significant association for any *E. coli* pathotype, indicate that molecular detection was not uniformly distributed across household matrices. The correlation between hands and surfaces was particularly strong, while the association between dishcloths and surfaces was also significant. These findings are consistent with the possibility of interconnected contamination pathways in which hands, cleaning materials, and frequently touched surfaces may participate in the movement of enteric bacteria within households [55,59]. However, the cross-sectional design cannot establish the direction of transfer or determine whether one matrix acted as the source of contamination. The correlations should therefore be interpreted as evidence of co-occurrence rather than demonstrated transmission.

The absence of the *E. coli* pathotype markers investigated, with the exception of ETEC-associated markers, in drinking-water samples is noteworthy. This finding suggests that drinking water was not a major detectable reservoir of the specific *Escherichia coli* virulence-associated markers assessed at the time of sampling. The detection of ETEC-associated markers indicates the presence of genetic material associated with this pathotype in a subset of drinking-water samples; however, molecular detection alone does not establish the presence of viable organisms, active toxin production, or an associated risk of disease. These findings should therefore be interpreted as evidence of detectable virulence-associated genetic material rather than confirmed occurrence of viable ETEC in household drinking water. Conversely, detection of multiple markers on household contact matrices despite their absence from water suggests that contamination may arise through sources or pathways other than the sampled drinking water.

The *Vibrio* findings presented a different pattern. Detection of *wbeO* and *sodB* was sporadic and occurred only in individual dishcloth, hand, and surface samples, with no significant association between environmental target and either marker. The absence of *ctxA* across all samples provides no molecular evidence of detectable cholera toxin gene carriage in the sampled household environment. The difference between the relatively frequent detection of *E. coli* pathotype markers and the limited detection of *Vibrio* markers may reflect differences in environmental occurrence, abundance, persistence, or the distribution of the specific targets investigated. The *Vibrio* results should nevertheless be interpreted cautiously because the low number of positive samples limits statistical power.

Molecular detection alone, however, cannot establish bacterial viability [60], culturability, or expression of virulence factors. Detection of *stx*, *eae*, *st*, *lt*, *eagg*, or *ial* demonstrates the presence of the target nucleic acid but does not establish that a viable pathogenic organism was present or that the associated virulence factor was expressed. Similarly, detection of *wbeO* or *sodB* should not be equated with confirmed viable or toxigenic *V. cholerae* without appropriate culture and confirmatory characterisation.

The findings therefore provide evidence of molecular contamination and potential opportunities for household exposure and cross-contamination, rather than direct evidence of infection or disease transmission. The particularly strong relationships involving hands, surfaces, and dishcloths nevertheless reinforce the importance of practical household hygiene measures, including effective handwashing, appropriate cleaning and drying of dishcloths, and regular cleaning of frequently touched surfaces. Future longitudinal studies incorporating culture-based confirmation, quantitative molecular approaches, strain characterisation, and assessment of temporal relationships between matrices would help establish the persistence, viability, directionality, and epidemiological significance of these environmental detections.

### Strengths and Limitations

This study provides preliminary evidence of enteric pathogen-associated genetic markers across multiple household environmental matrices, including drinking water, dishcloths, hands, household surfaces and toilet seats. However, several limitations should be considered. Voluntary convenience sampling may have introduced selection bias, as participating households may differ from non-participating households in socioeconomic characteristics, hygiene practices, environmental conditions and health awareness. The relatively small sample size and cross-sectional design further limit statistical power, generalisability and the ability to infer temporal or causal relationships. The findings should therefore be regarded as exploratory rather than representative of the wider population.

The absence of clinical disease surveillance and a structured hygiene questionnaire prevented assessment of associations between environmental contamination, household practices (including hand hygiene, dishcloth use, food preparation, cleaning and water-handling behaviours), and diarrhoeal illness. Similarly, potential within-household clustering may have reduced the independence of observations. Furthermore, PCR-based detection identifies target genetic material but does not establish organism viability, infectivity or clinical causation. The absence of culture-based isolation and viability assessment therefore limits conclusions regarding the presence of viable pathogenic organisms and potential transmission.

Although PCR provides sensitive, specific and rapid detection, its application in low-resource settings may be constrained by infrastructure and cost. Alternative approaches, including loop-mediated isothermal amplification (LAMP), recombinase polymerase amplification (RPA), rapid antigen-based assays and paper-based molecular platforms, may offer more accessible options for field-based surveillance. Future studies should incorporate larger, randomly selected, longitudinally sampled populations, together with structured hygiene assessments, clinical surveillance, culture-based isolation and viability, or genomic characterisation, to better establish the persistence, plausible faecal-oral transmission routes and public-health significance of DEC-associated contamination in household environments.

## 5. Conclusions

This study identified substantial molecular evidence of diarrhoeagenic *E. coli* markers across household environmental matrices, with dishcloths, hands, and household surfaces showing the greatest diversity and frequency of detection. The environmental target was significantly associated with detection of several *E. coli* pathotypes and with overall pathotype detection, while strong correlations between hands, surfaces, and dishcloths suggest interconnected household contamination patterns. In contrast, *Vibrio*-associated markers were detected only sporadically, with no significant matrix-specific associations and no detection of the *ctxA* cholera toxin gene.

Collectively, these findings indicate that household contact matrices may represent important sites of molecular contamination and potential cross-contamination. The results support the importance of household hygiene interventions targeting hands, dishcloths, and frequently touched surfaces. However, because the study relied on molecular detection and did not establish viability, infectivity, toxin expression, or transmission, the findings cannot demonstrate that the detected markers represented viable infectious organisms or caused human disease. Culture-based confirmation and longitudinal epidemiological studies are needed to determine the public-health significance of these household environmental detections.

## Figures and Tables

**Table 2:** Microbial Quality of Drinking Water in Zandspruit, expressed as MPN/100 mL.

Site	Indicator Organism	0 MPN	1–10 MPN	11–100 MPN	>100 MPN
**Zandspruit**	**Total Coliforms**	26	2	1	0
	***E. coli***	29	0	0	0

**Table 3:** Frequency table showing the positive/screened (%) of *E. coli* associated genetic markers in household environmental samples.

Target	Water	Dishcloth	Hand	Surfaces	Toilet
STEC (*stx1* and/or *stx2*)	0/29 (0.0%)	6/28 (21.4%)	10/29 (34.5%)	9/28 (32.1%)	6/29 (20.7%)
Typical EPEC (*eae^+^ bfp^+^*, non-STEC)	0/29 (0.0%)	4/28 (14.3%)	0/29 (0.0%)	0/28 (0.0%)	0/29 (0.0%)
Atypical EPEC (*eae^+^ bfp^−^*, non-STEC)	0/29 (0.0%)	2/28 (7.1%)	3/29 (10.3%)	2/28 (7.1%)	6/29 (20.7%)
ETEC (*st* and/or *lt*)	5/29 (17.2%)	12/28 (42.9%)	9/29 (31.0%)	10/28 (35.7%)	1/29 (3.4%)
EAEC (*eagg*)	0/29 (0.0%)	6/28 (21.4%)	6/29 (20.7%)	6/28 (21.4%)	5/29 (17.2%)
EIEC (*ial*)	0/29 (0.0%)	4/28 (14.3%)	0/29 (0.0%)	1/28 (3.6%)	2/29 (6.9%)
*astA*-positive *E. coli*	0/29 (0.0%)	8/28 (28.6%)	5/29 (17.2%)	5/28 (17.9%)	2/29 (6.9%)

**Table 4:** Chi-square/exact test of association (matrix × detection) for *E. coli.*

Target	χ² (df = 4)	Exact MC p	BH-adj p	Cramér’s V
STEC (*stx1* and/or *stx2*)	12.65	0.0113	0.0245	0.297
Typical EPEC (*eae*^+^ *bfp*^+^, non-STEC)	16.90	0.0028	0.0091	0.344
Atypical EPEC (*eae*^+^ *bfp*^−^, non-STEC)	7.93	0.0843	0.1218 (ns)	0.236
ETEC (*st* and/or *lt*)	14.76	0.0040	0.0104	0.321
EAEC (*eagg*)	7.23	0.1241	0.1614 (ns)	0.225
EIEC (*ial*)	8.64	0.0409	0.0665 (ns)	0.246
*astA*-positive *E. coli*	11.48	0.0178	0.0331	0.283

ns = not significant after Benjamini–Hochberg correction. Exact MC p = Monte Carlo Fisher–Freeman–Halton-equivalent exact *p*-value (10,000 simulations).

**Table 5:** Spearman correlation between matrix contamination profiles for *E. coli*.

Matrix A	Matrix B	Spearman ρ	*p*-Value
Water	Dishcloth	0.62	0.135
Water	Hand	0.41	0.358
Water	Surfaces	0.61	0.144
Water	Toilet	−0.42	0.354
Dishcloth	Hand	0.68	0.094
Dishcloth	Surfaces	0.76	0.046 *
Dishcloth	Toilet	−0.28	0.546
Hand	Surfaces	0.95	0.001 *
Hand	Toilet	0.39	0.381
Surfaces	Toilet	0.27	0.554

Based on *n* = 7 paired pathotype-prevalence values per matrix pair. * *p* < 0.05. Hand–Surfaces (ρ = 0.95, *p* = 0.001) and Dishcloth–Surfaces (ρ = 0.76, *p* = 0.046) are statistically significant, indicating these matrices share a similar pathotype profile—consistent with a shared contamination pathway (e.g., hand-to-surface transfer).

**Table 6:** Frequency table showing the positive/screened (%) of *Vibrio* spp. associated genetic markers in household environmental samples.

Target	Water	Dishcloth	Hand	Surfaces	Toilet
Serogroup O1 (*wbeO*)	0/29 (0.0%)	1/28 (3.6%)	0/29 (0.0%)	0/28 (0.0%)	0/29 (0.0%)
Species-specific (*sodB*)	0/29 (0.0%)	0/28 (0.0%)	1/29 (3.4%)	1/28 (3.6%)	0/29 (0.0%)
Cholera toxin (*ctxA*)	0/29 (0.0%)	0/28 (0.0%)	0/29 (0.0%)	0/28 (0.0%)	0/29 (0.0%)

**Table 7:** Chi-square/exact test of association (matrix × detection) for *Vibrio* spp.

Target	χ^2^ (df = 4)	Exact MC p	BH-adj p	Cramér’s V
Sero group O1 (*wbeO*)	4.14	0.3866 (ns)	0.4569 (ns)	0.170
Species-specific (*sodB*)	3.06	0.7534 (ns)	0.7534 (ns)	0.146
Cholera toxin (*ctxA*)	—	—	—	—

Chi-square/exact test results for *Vibrio* markers. None of the individual pathogenic markers reaches significance after correction (too few positives).

## Data Availability

The original contributions presented in this study are included in the article. Further inquiries can be directed to the corresponding author.
